# Epstein-Barr Virus-induced Jaundice

**DOI:** 10.5811/cpcem.2019.10.45049

**Published:** 2020-01-21

**Authors:** Jessica Herold, Felipe Grimaldo

**Affiliations:** Naval Medical Center San Diego, Department of Emergency Medicine, San Diego, California

## Abstract

Infectious mononucleosis is primarily caused by Epstein-Barr virus (EBV) and is a common diagnosis made in emergency departments worldwide. Subclinical and transient transaminase elevations are a well-established sequela of EBV. However, acute cholestatic hepatitis is a rare complication. EBV infection should be considered as part of the differential diagnosis in patients with an obstructive pattern on liver function tests without evidence of biliary obstruction demonstrated on advanced imaging.

## INTRODUCTION

Epstein-Barr virus (EBV) is a DNA virus that causes infectious mononucleosis. Infections are common in childhood and young adulthood but are usually asymptomatic. The classic triad of fever, tonsillitis, and lymphadenopathy that frequently presents to the emergency department (ED) is more common in adolescents and adults. Elevations in liver transaminases are not uncommon with EBV infection, but are usually transient and rarely progress to fulminant hepatitis.^1^,^2^ However, significant cholestasis and jaundice are rare complications with an incidence of less than 5%.^3^,^4^,^5^

## CASE REPORT

A 23-year-old previously healthy male presented to the ED with complaints of a headache that was gradual in onset and had been present for the prior 24 hours. He noted some lightheadedness and dizziness while standing, which prompted him to present to the ED for evaluation. He was febrile to 100.5 degrees Fahrenheit (F) and tachycardic to 110 beats per minute (bpm). The remainder of his physical exam was grossly unremarkable with no meningeal signs or focal neurologic deficits. He was provided antipyretics and intravenous (IV) fluids with complete resolution of his symptoms and discharged home with a diagnosis of viral syndrome.

Two days later, he returned to the ED with complaints of continued headache and fever. He recalled a “dry, tickling throat,” which was brief and self-limited in the prior two days. He was tachycardic, but afebrile on exam. With the exception of his tachycardia, his physical exam was again unremarkable without an identifiable infectious source. Laboratory evaluation demonstrated a bandemia of 8% (reference range 0–5) as well as mild transaminitis with alanine aminotransferase (ALT) 177 units per liter (U/L) (reference range 17–63) and aspartate aminotransferase (AST) 171 U/L (reference range 12–39). His rapid heterophile antibody test was positive. He was discharged home with precautions to avoid contact sports and to have repeated liver function tests performed by his primary care provider.

Three days after his second ED visit, he returned with jaundice, dark urine, and with continued fever and fatigue. He denied sore throat, cough, chest pain, abdominal pain, vomiting, diarrhea, hematuria, dysuria, or rash. He was again febrile with a temperature of 100.9° F and a pulse rate of 109 bpm. There was noticeable scleral icterus and diffuse jaundice. He was also noted to have multiple, palpable, posterior cervical lymph nodes.

Laboratory evaluation was notable for a leukocytosis of 14.8 ×10^3 cells per microliter (mcL) (reference 4.0–10.5) with lymphocytic predominance of 24% and thrombocytopenia of 99×10^3 cells/mcL (reference range 150–450). Comprehensive metabolic panel was notable for mild hyponatremia of 133 millimoles (mmol) per L (reference range 136–145 mmol/L), total bilirubin of 7.93 milligrams per deciliter (mg/dL) (reference 0.15–1.00), direct bilirubin of 6.9 mg/dL (reference range <0.2–0.3), alkaline phosphatase of 198 U/L (reference range 40–129), ALT of 753 U/L (reference range 17–63), and AST 692 U/L (reference range 12–39). Coagulation studies were within normal limits. Acetaminophen level was negative at <1.5 micrograms per milliliter (reference range 10–30). Hepatitis serologies were notable for a reactive hepatitis B virus core antibody, non-reactive hepatitis B core antibody IgM, positive hepatitis B surface antibody, and negative hepatitis B surface antigen consistent with immunity due to natural infection. Hepatitis C antibody was non-reactive. Human immunodeficiency virus testing was negative. Blood cultures were also negative. EBV heterophile antibodies were positive.

A formal right upper quadrant ultrasound demonstrated a mildly enlarged liver with normal contour. The gallbladder was visualized and noted to be contracted. The gallbladder wall was noted to be mildly thickened with a measurement of 0.34 centimeters. There was no evidence of cholelithiasis.

The patient was admitted to the hospital for supportive care and further laboratory evaluation. He was provided IV fluids, and liver function tests were trended every six hours. Liver enzymes gradually decreased and his jaundice resolved. His thrombocytopenia was thought to be related to acute hepatitis. Coagulation studies remained within normal limits. He was discharged from the hospital with a diagnosis of cholestatic hepatitis secondary to EBV. He followed up with internal medicine and had serial liver function tests over the subsequent weeks.

## DISCUSSION

EBV is a member of the herpes virus family and has a seroprevalence of 90–95% worldwide.^6^ EBV is the primary cause of infectious mononucleosis, which can occur at all ages but is most common during adolescence and early adulthood.^7^,^8^ The likelihood of symptomatic disease is higher in older populations, and the risk of severe symptoms is positively correlated with age.^7^ Classic clinical symptoms of infectious mononucleosis include a triad of fever, pharyngitis, and adenopathy, which usually manifest after an incubation period of four to seven weeks.^3^,^7^ EBV is transmitted through saliva and infiltrates epithelial cells and resting B-cells, replicating and spreading throughout the body.^9^ After its incubation period, activation of cytotoxic T lymphocytes and natural killer cells occurs, leading to a cell-mediated immune response.^7^,^10^ Most infections are self-limited with an overall excellent prognosis; however, in some cases EBV infection complications can range from mild hepatitis to lymphoproliferative disorders, hepatosplenomegaly and, rarely, acute liver failure.^8^

The diagnosis of EBV is made based on clinical presentation and laboratory findings. Clinical presentation may include the classic triad of symptoms as well as tonsillar exudates, palatal petechiae, hepatosplenomegaly, and hepatitis.^9^,^11^ About 5–10% may present with jaundice.^7^ Laboratory analysis may demonstrate an absolute lymphocytosis in which more than 10% of the cells are atypical and positive heterophile antibody titers. Heterophile antibody titers may be falsely negative in the first week and may be consistently negative in approximately 10% of patients. If this is the case, EBV viral capsid antigen IgG and IgM antibody tests as well as EBV nuclear antigen antibodies may be helpful in distinguishing the infection.^7^,^8^ Serum aminotransferase levels are usually elevated by less than five times the upper limit of normal and rarely reach over 1000 U/L.

**CPC-EM Capsule**

What do we already know about this clinical entity?

Infectious mononucleosis is primarily caused by Epstein-Barr virus and is a common diagnosis in the emergency department.

What makes this presentation of disease reportable?

Cholestatic hepatitis is a rare presentation of Epstein-Barr virus with only approximately 5% of patients presenting with jaundice.

What is the major learning point?

As emergency providers, it is important that our differential diagnoses include Epstein-Barr virus when evaluating patients with cholestasis.

How might this improve emergency medicine practice?

Keeping Epstein-Barr virus on the differential as a cause of cholestatic hepatitis may save money on further diagnostic imaging and work up.

Liver tissue injury is common in EBV infection with about 75% of patients exhibiting an increase in aminotransferases.^7^ Cholestatic hepatitis is a rare sequelae of EBV, and jaundice is seldom reported; however, it is more frequent in people aged 35 and older.^12^,^13^,^14^ If seen, jaundice may be due to autoimmune hemolytic anemia, cholestasis due to acalculous cholecystitis, biliary duct obstruction due to abdominal lymphadenopathy, or cholestatic hepatitis.^9^,^15^ The pathogenesis of cholestatic hepatitis due to EBV is unclear. EBV unlikely infects hepatocytes, biliary epithelium or vascular endothelium.^3^ It has been considered that cholestasis may be related to lipid peroxidation and consequent free radical production.^9^,^12^

Once the diagnosis of EBV is made, further workup is generally not warranted.^1^,^2^ Treatment of uncomplicated infectious mononucleosis usually requires only symptomatic treatment with antipyretics, hydration, and rest.^1^,^2^,^6^ Often patients may experience abdominal pain, which may in part be due to splenomegaly.^6^ If splenomegaly is present, it is prudent to warn the patient against physical activity to prevent splenic rupture; although rare, it has an incidence of 0.1–0.2%.^10^ Rare complications of EBV may include upper airway obstruction, peritonsillar abscess, encephalitis, myocarditis, or pleural effusion.^8^,^9^,^10^ The patient in this case recovered without serious intervention.

## CONCLUSION

EBV infection has a high prevalence throughout the world. Although EBV often leads to self-limited infectious mononucleosis, it should be considered in the diagnosis of cholestatic hepatitis even in the absence of typical infectious mononucleosis clinical signs. It is important for emergency care providers to expand their differential to include EBV when working up a patient with cholestasis. Heterophile antibody testing is widely available and quick to perform and may be useful for rapid indication of EBV hepatitis. Providers should consider the diagnosis of EBV in all patients with unexplained hepatitis regardless of their age or symptomatology.
